# Integrating machine learning into acupuncture research: a scoping review

**DOI:** 10.3389/fneur.2025.1689061

**Published:** 2026-01-14

**Authors:** Wancy Chan, Guan-Jun Huang, Chien-Chen Huang, Yi-Hung Chen

**Affiliations:** 1 Graduate Institute of Acupuncture Science, China Medical University, Taichung, Taiwan; 2 Chinese Medicine Research Center, China Medical University, Taichung, Taiwan; 3 Department of Chinese Medicine, An Nan Hospital, China Medical University, Tainan, Taiwan; 4 School of Post-Baccalaureate Chinese Medicine, College of Chinese Medicine, China Medical University, Taichung, Taiwan; 5 The International Master Program in Integrative Health, China Medical University, Taichung, Taiwan; 6 Office of Research and Development, Asia University, Taichung, Taiwan

**Keywords:** acupuncture, artificial intelligence, efficacy prediction, machine learning, pain

## Abstract

**Objectives:**

Machine learning offers new tools to address the variability and subjectivity in acupuncture research. This scoping study aims to map existing literature on the use of machine learning in acupuncture research. It identifies the disease conditions most frequently targeted for machine learning-based efficacy prediction, examines the machine learning methods employed, and assesses the data inputs, methodological limitations, and existing knowledge gaps in the field.

**Methods:**

We conducted a comprehensive literature search in PubMed database using the keywords “acupuncture” and “machine learning” for publications from 2011 to 2024.

**Results:**

A total of 36 relevant articles were identified, with a notable increase after 2019. Most publications originated from China. Seventeen studies focused on predicting acupuncture efficacy, primarily for pain-related conditions. The remaining studies addressed diverse topics, including acupuncture manipulation technique detection, prescription recommendation, exploration of efficacy-related factors, acupoint sensitization prediction and specificity identification, acupuncture usage frequency prediction, investigation of acupoint–meridian conduction effects, and acupuncture robotic point localization. In efficacy prediction studies, support vector machines were the most frequently employed algorithm. Seven of the 11 studies combined Magnetic Resonance Imaging as a feature for their models, and treatment responder classification was often used as labels.

**Conclusion:**

Most studies reported encouraging predictive performance, indicating that machine learning methods can be effectively applied to acupuncture efficacy prediction. Support vector machines, in particular, demonstrated significant potential. These findings suggest that machine learning could improve the precision and efficiency of acupuncture treatments and help create more personalized and effective treatment plans. However, small sample sizes, methodological heterogeneity, inconsistent data types, and lack of standardized datasets limit model generalizability and comparability. Important gaps remain, including mechanistic understanding, long-term outcome prediction, and evaluation of clinical impact. Future research should focus on larger, multi-center studies with standardized protocols, rigorous external validation, and assessment of clinical utility to advance the integration of machine learning into acupuncture practice.

## 1 Introduction

Artificial intelligence (AI) has been widely used in numerous fields. Its introduction changes the way data are analyzed and how decisions are made. Machine learning (ML) plays an important role in the advancement of AI. ML enables systems to learn and improve from data using algorithms, without requiring explicit programming for every task (1). ML algorithms can analyze large datasets to identify patterns, make predictions, or classify information (2). ML also offers extensive feature selection diversity, allowing for the incorporation of many data types in analysis. It can handle structured and unstructured information, covering demographic details, laboratory results, medical images, and patient records. ML is applied in several areas of medicine, such as risk assessment, diagnostic support, and treatment outcome prediction (3). Acupuncture has been a core part of traditional Chinese medicine (TCM) for over 2,500 years. It involves the stimulation of acupoints, which are located at specific sites of the human body, by insertion of fine metal needles, followed by manipulation (4). Since the 1970s, acupuncture has rapidly gained popularity in Western countries (5). In 1979, the World Health Organization (WHO) recommended it for 43 diseases (6). A 1998 National Institutes of Health consensus conference confirmed its efficacy for postoperative and chemotherapy-induced nausea and vomiting, as well as dental pain (7). Besides, acupuncture is beneficial as an adjunct or alternative treatment for conditions like addiction, stroke rehabilitation, headaches, menstrual cramps, tennis elbow, fibromyalgia, myofascial pain, osteoarthritis, low back pain, carpal tunnel syndrome, and asthma (7). In 2002, a WHO review of 225 clinical trials concluded that acupuncture was effective for 28 diseases and beneficial for 63 others (8). Despite its long history and widespread use, the underlying mechanisms of acupuncture remain unclear, and individual responses to treatment can vary. Their practical application often relies on the practitioner’s subjective judgments in diagnosis, prognosis, and technique evaluation.

In this context, integrating ML into acupuncture research has become increasingly relevant in recent years. By providing objective prediction models and identifying patterns in complex datasets, ML can aid in overcoming the uncertainty in acupuncture treatment outcomes, which are often influenced by subjective practitioner assessments. Moreover, ML methods can be used to explore the therapeutic mechanisms of acupuncture, which remain incompletely understood.

This scoping review aims to map the existing literature on ML applications in acupuncture research. We identify the disease conditions most frequently targeted for ML-based efficacy prediction, examine the ML methods employed, and assess the data inputs, methodological limitations, and knowledge gaps in the field.

## 2 Methods

To conduct this scoping review, we carried out a search on PubMed from 2011 to 2024 using the terms “acupuncture” and “machine learning.” Articles were considered eligible if they met the following criteria: (1) the study focused on acupuncture, (2) ML methods were applied, and (3) results were reported in relation to acupuncture research. Articles were excluded if they did not involve acupuncture directly, did not use ML, or were unrelated to the scope of this review. Screening and selection were performed to ensure relevance, with uncertainties resolved through discussion.

## 3 Results

**Figure 1:**
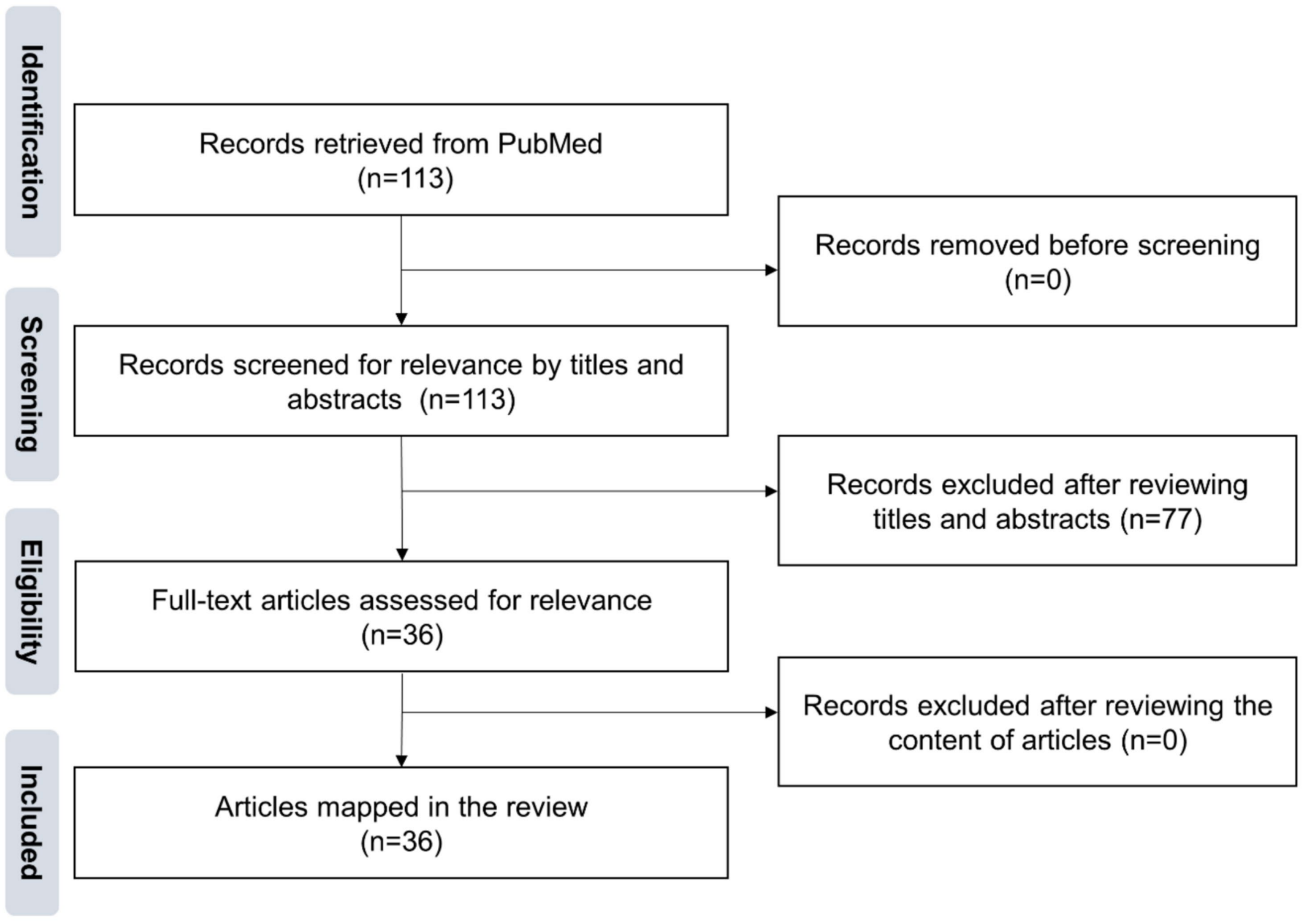
Literature screening process.

A total of 113 articles were initially retrieved. After screening, 36 articles were considered relevant. The article selection process is summarized in Figure 1. Among these, 17 articles focused on ML prediction of acupuncture efficacy. Another 11 articles explored various other applications of ML in acupuncture research. The remaining eight articles were literature reviews discussing the use of ML in the context of acupuncture.

### 3.1 Publication trends over time

From 2011 to 2018, only one relevant article was published in each of the years 2011, 2013, 2014, 2015, and 2018, indicating limited research in this area during that period. Starting in 2019, there was a notable increase in the number of publications, with two articles published that year. This upward trend continued in the subsequent years, with the number of published articles rising to 6 in 2020, 5 in 2021, and 9 in 2022. However, this was followed by a significant decline over the next 2 years: 6 in 2023 and 3 in 2024, as illustrated in Supplementary Figure 1.

### 3.2 Geographic distribution of studies

From a country/region perspective, the primary contributor to research on these topics is China. Specifically, out of the 36 articles, 31 originated from China, 3 from the United States, 1 from South Korea, and 1 from Hong Kong SAR, as shown in Supplementary Figure 2.

### 3.3 Journal distribution of publications

Regarding publication venues, the journals that have published the greatest number of relevant articles include *Frontiers in Neurology* and *Chinese Acupuncture & Moxibustion*, each publishing four articles on ML and acupuncture research. *Acupuncture Research* has published three articles. *Chinese Medicine, Computational and Mathematical Methods in Medicine*, and *Frontiers in Neuroscience* have each published two articles. Nineteen other journals have published only one article each, as shown in Table 1.

**Table 1:** Journals publishing research on machine learning applications in acupuncture.

Journal	Number
Frontiers in Neurology	4
Chinese Acupuncture & Moxibustion	4
Acupuncture Research	3
Chinese Medicine	2
Computational and Mathematical Methods in Medicine	2
Frontiers in Neuroscience	2
AMIA Annual Symposium Proceedings	1
Annual International Conference of the IEEE Engineering in Medicine and Biology Society	1
Cerebral Cortex	1
EPMA Journal	1
Evidence-Based Complementary and Alternative Medicine	1
Frontiers in Aging Neuroscience	1
Frontiers in Molecular Neuroscience	1
Frontiers in Oncology	1
Human Brain Mapping	1
IEEE Transactions on Neural Systems and Rehabilitation Engineering	1
International Journal of Data Mining and Bioinformatics	1
Journal of Psychiatric Research	1
Magnetic Resonance Imaging	1
Mathematical Biosciences and Engineering	1
Medical Acupuncture	1
Medicine (Baltimore)	1
Neural Plasticity	1
NeuroImage: Clinical	1
The Scientific World Journal	1

### 3.4 The diverse applications of machine learning in acupuncture research

**Figure 2:**
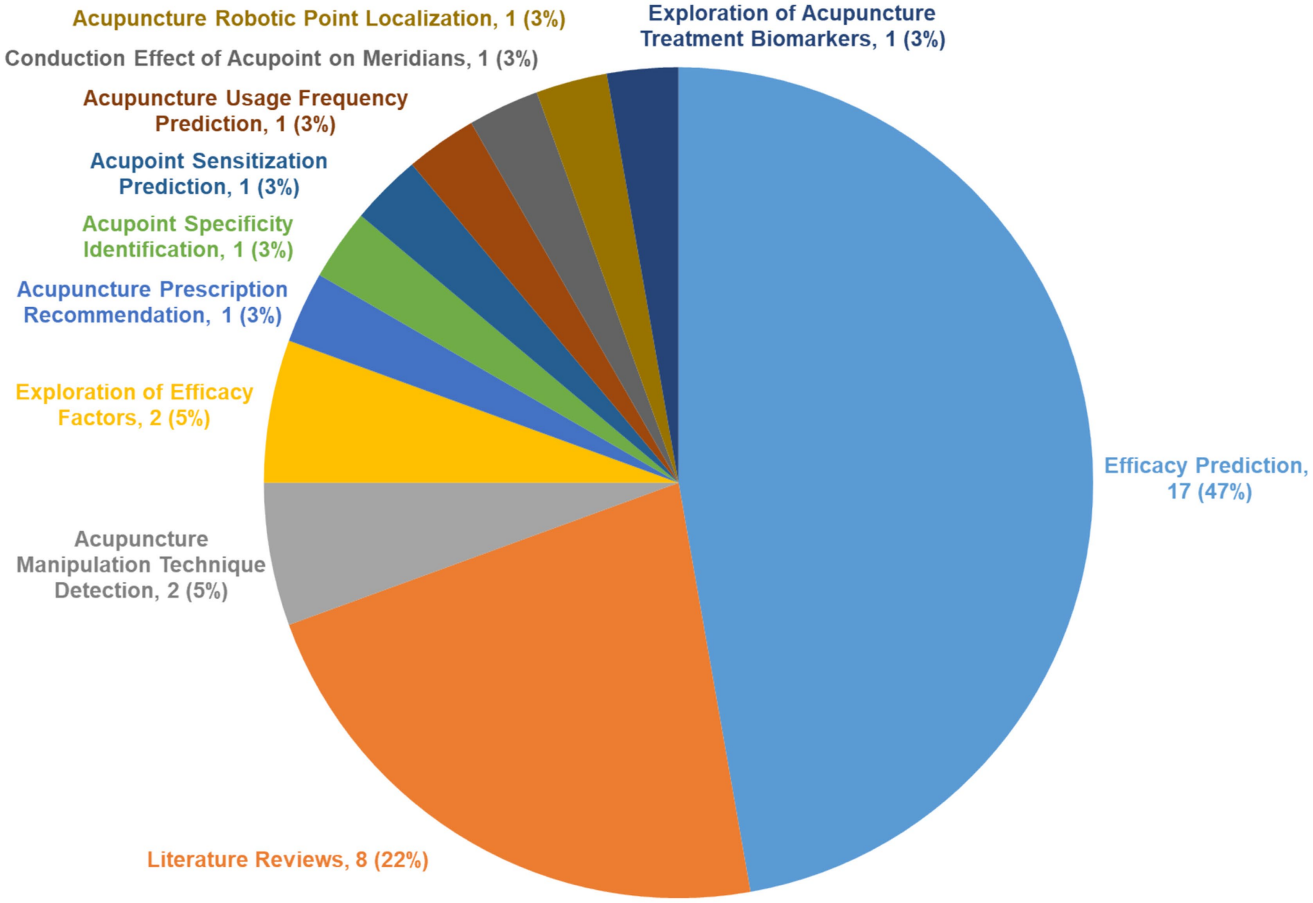
Research purposes of machine learning in acupuncture.

Analysis of the 36 included articles and examination of research objectives identified several applications. Applications of ML in acupuncture research are shown in Figure 2:

- Efficacy Prediction: 17 articles focus on predicting the efficacy of acupuncture treatments.
- Literature Reviews: eight articles provide in-depth reviews of existing literature and methodologies.
- Acupuncture Manipulation Technique Detection: two articles investigate the detection and classification of acupuncture techniques using ML algorithms.
- Exploration of Efficacy Factors: two articles examine the factors influencing the efficacy of acupuncture treatments.

Furthermore, seven articles address additional objectives:

- Acupuncture Prescription Recommendation: Exploring ML models for recommendation of personalized acupuncture prescriptions.
- Acupoint Sensitization Prediction: Predicting the accuracy of sensitization at acupoints following treatment.
- Acupoint Specificity Identification: Demonstrating the specificity of different acupoints in treatment outcomes.
- Acupuncture Usage Frequency Prediction: Studying differences in acupuncture usage frequency among users in some regions.
- Conduction Effect of Acupoint on Meridians: Identifying factors that influence the propagated sensation along Stomach meridians at Zusanli (ST36).
- Acupuncture Robotic Point Localization: Developing acupuncture robots in acupoint localization.
- Exploration of Acupuncture Treatment Biomarkers: Exploring the use of exosomal miRNAs as biomarkers in migraine diagnosis and treatment.

### 3.5 Pain as the primary focus in efficacy prediction studies

**Figure 3:**
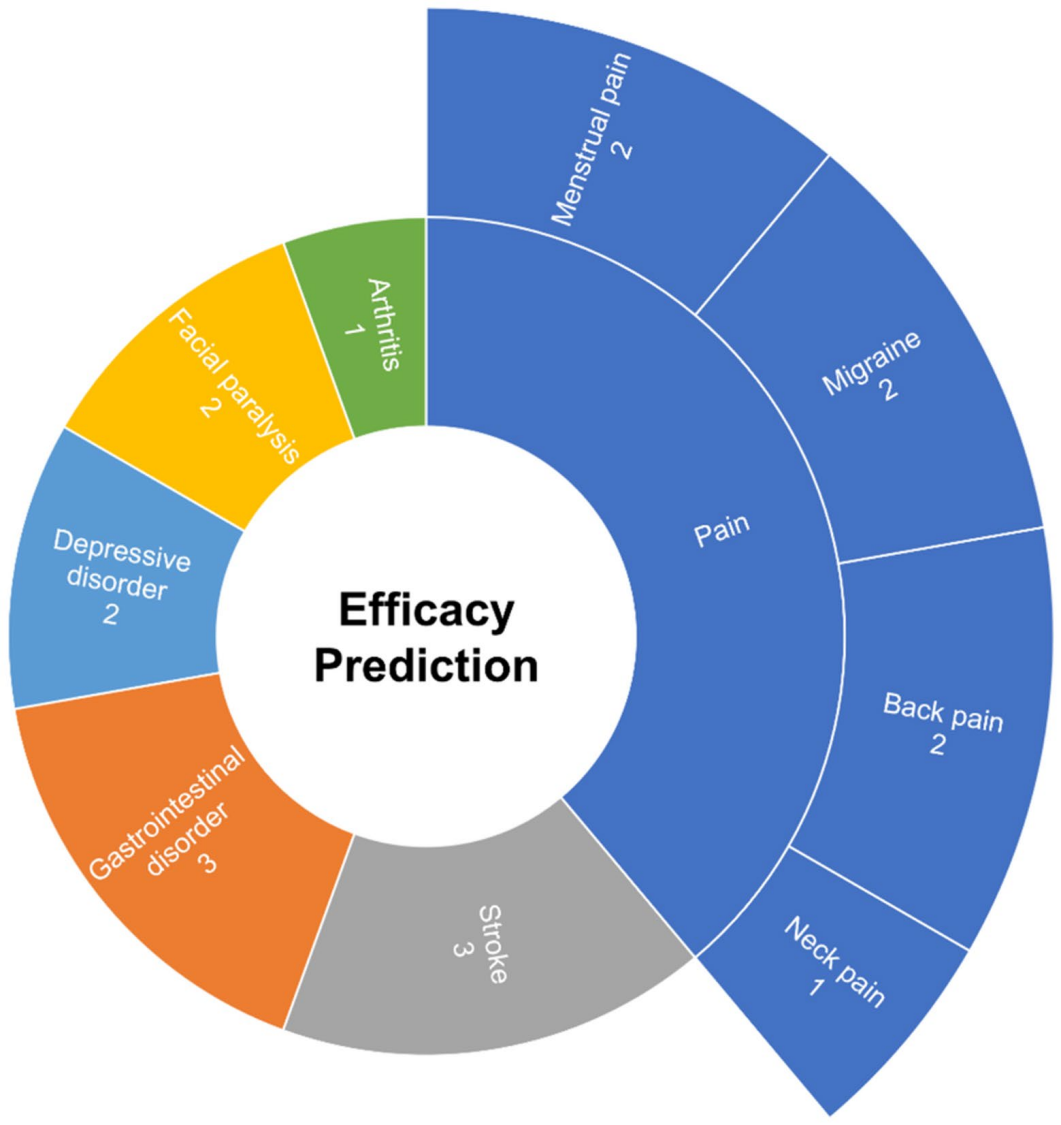
Focus areas within efficacy prediction research.

Each of the 17 articles on acupuncture prediction has a distinct research focus. Pain, addressed in six articles, is the most common focus for efficacy prediction. Following pain, there are three articles each on stroke and gastrointestinal disorders, two articles each on facial paralysis and depression, and 1 on arthritis. Further analysis reveals that pain treatment includes various conditions that involve back pain, neck pain, migraines, and menstrual pain, as shown in Figure 3.

### 3.6 Machine learning in acupuncture efficacy prediction

There are four commonly used learning methods in ML: supervised, unsupervised, semi-supervised and reinforcement learning (9). Deep learning is a subset of ML, and is often discussed with these methods.

Supervised learning uses known input and output data to train models, allowing it to predict outputs for new data (10). Its common tasks are classification and regression (9). Most of the algorithms in our study, such as support vector machine, random forest and logistic regression, fall under supervised learning.

Unsupervised learning analyzes and clusters unlabeled data by algorithms, its main tasks are clustering, pattern discovery and dimensionality reduction (11). Fuzzy C-Means clustering is one representative method.

Semi-supervised learning trains models using both labeled and unlabeled data (12). It uses abundant unlabeled data to improve performance when labeled data is limited (13).

Reinforcement learning takes a different approach. It learns by interacting with an environment and selecting actions that maximize rewards (14). Deep Q-Network (DQN) is one of the widely used algorithms (15).

Deep learning is based on neural networks with many layers (16). It performs well with high-dimensional data and is applied in many fields, including science, business, and government (17). Convolutional Neural Networks mentioned in our study are one of the examples of deep learning models.

**Figure 4:**
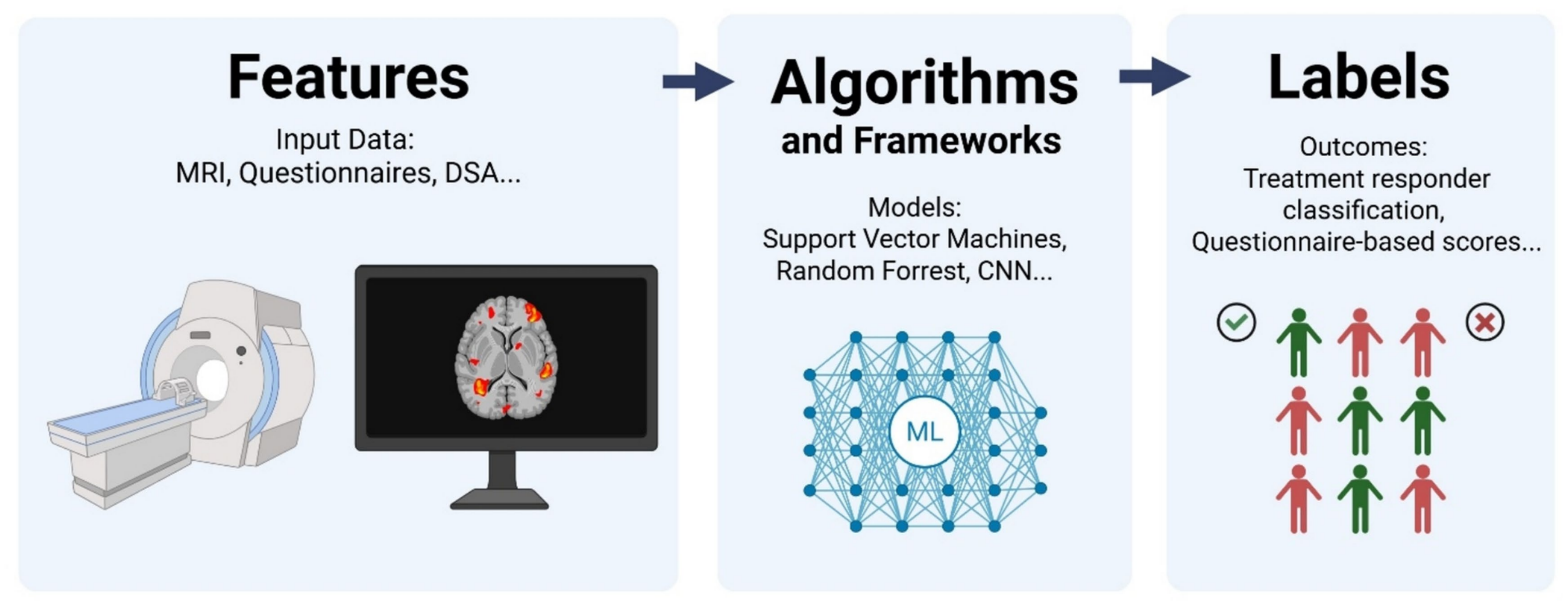
Relationship between features, algorithm, and labels in treatment efficacy prediction (Created in BioRender).

In ML, there are primarily two components: data (features and labels) and algorithms. Features are the input variables, while labels represent the outcomes to be predicted. For instance, diagnostic images like chest X-rays may be used as features, with treatment efficacy serving as the label. By inputting a large amount of participant data, the algorithm can predict the label based on the features of other samples. This predictive capability defines the role of the algorithm component in the ML process. The relationship between features, algorithms, and labels is shown in Figure 4.

Studies may adopt different ML algorithms and feature-label pairs, however, the underlying principle remains consistent: using algorithms to analyze features and derive labels. Features in acupuncture research can range from imaging and questionnaire data to medical records and tactile sensor inputs. Similarly, labels can be defined based on specific criteria; in acupuncture efficacy prediction, labels could be “effective” or “ineffective” based on predefined outcomes.

Given the focus on efficacy prediction in acupuncture research and its potential to enhance medical efficiency, Table 2 presents studies on ML prediction.

**Table 2:** Summary of research on machine learning and efficacy prediction.

Ref.	Conditions	Features	Algorithms	Types of ML	Labels	Findings
(28)	Chronic low back pain	Functional connectivity features from functional MRI	Support vector regression	Supervised learning	Pain outcome (Visual Analogue Scale)	Explained 34% (real) and 29% (sham) of pain variance
(37)	Chronic low back pain	Baseline clinical phenotype, Roland Morris Disability Questionnaire score (RMDQ)	Least absolute shrinkage and selection operator	Supervised learning	RMDQ scores change	Predicted 40% of outcome variance in functional disability scores
(29)	Migraine	Z-Transformed Amplitude of Low-Frequency Fluctuation Maps	Support vector classification, Support vector regression	Supervised learning	Pain score, headache days	*R*^2^ of 0.38 for VAS, *R*^2^ of 0.28 for migraine days
(22)	Migraine	Gray matter volume in MRI	Support vector machine	Supervised learning	Responder/non-responder	83% accuracy; responders showed volume increase in key brain regions
(36)	Neck pain	Symptom scores (Northwick Park Neck Pain Questionnaire, McGill Pain Questionnaire, SF-36 questionnaire for Quality of Life)	Similarity-based learning framework	Supervised/Unsupervised learning	Pain relief classification	Achieved 73.8% accuracy; ensemble learning boosted performance
(27)	Primary dysmenorrhea	Resting-state functional connectivity (FC) from MRI, pain score	Support vector regression	Supervised learning	Pain score change	Baseline FC predicted Visual Analogue Scale reduction; different effects for real vs. sham
(25)	Stroke	Functional MRI features of brain motor areas	Support vector machine	Supervised learning	Motor function improvement scores	75.5% accuracy; identified 8 key regions related to motor improvement
(34)	Stroke	Digital Subtraction Angiography imaging, clinical scores	YOLOX, faster Region-based Convolutional Neural Networks, TOOD, random forest	Deep Learning/ Supervised Learning	Responder/non-responder	93.6% accuracy; Internal Carotid Artery and Middle Cerebral Artery stenosis most predictive of treatment response
(26)	Stroke	Digital Subtraction Angiography stenosis segments, demographics	AutoGluon framework (random forest, ExtraTrees, k-Nearest Neighbors, linear regression, logistic regression, polynomial regression, ridge regression, support vector machine, decision tree, AdaBoost)	Supervised Learning	Treatment effectiveness classification	Prediction accuracy: NIHSS 84.3%, FMA-UE 77.8%, MBI 88.1%
(33)	Facial paralysis	Blood flow image features (LASCA), electromyogram	Convolutional neural networks	Deep Learning/ Supervised Learning	Prognosis Outcomes	Predicted recovery trends accurately
(32)	Facial paralysis	Electromyography (EMG)	Multi-view convolutional neural networks	Deep Learning/ Supervised Learning	Responder classification	Multi-view CNN reached 88.2% accuracy; better than single-view for EMG
(31)	Functional dyspepsia	Clinical features (symptoms, diagnosis)	Support vector classification (SVC), Support vector regression (SVR)	Supervised learning	Treatment responder status, continuous outcome prediction	SVC: Accuracy 0.773; SVR: *R*^2^ = 0.446 (NDLQI), 0.413 (NDSI)
(21)	Functional dyspepsia	Functional MRI connectivity features	Support vector machine	Supervised learning	Responder/non-responder, symptom change	76% accuracy in classification; *R*^2^ of 0.24 in predicting symptoms relief; 38 brain network features distinguished responders
(30)	Functional dyspepsia	Resting-state functional connectivity (FC) from MRI, clinical data	Support vector classification, Support vector regression	Supervised learning	Responder status, symptom score change	SVC accuracy 88% in predicting responder; *R*^2^ of 0.453 in predicting symptoms; confirmed robustness in validation cohort
(23)	Depression	Electroencephalogram	Logistic regression, random forest, support vector machine	Supervised learning	Treatment responder status	84.6% accuracy in classifying responders
(24)	Major Depressive Disorder	Clinical data	XGBoost, support vector machine, logistic regression, random forest, k-nearest neighbor	Supervised learning	Responder/non-responder	XGBoost best accuracy 73%; anxiety score, BMI and traditional Chinese medicine syndromes as top predictors
(35)	Knee osteoarthritis	CT imaging	Convolutional neural networks	Deep Learning/ Supervised Learning	Treatment effectiveness classification	CT showed improved bone/cartilage metrics; better clinical scores in test group

CNN, convolutional neural networks; CT, computed tomography; EMG, electromyography; FC, functional connectivity; FMA-UE, Fugl-Meyer Assessment for Upper Extremity; LASCA, Laser Speckle Contrast Analysis; MBI, modified Barthel Index; ML, machine learning; MRI, magnetic resonance imaging; NDLQI, Nepean Dyspepsia Life Quality Index; NDSI, Nepean Dyspepsia Symptom Index; NIHSS, National Institute of Health Stroke Scale; RMDQ, Roland Morris Disability Questionnaire; SVC, support vector classification; SVR, support vector regression; VAS, Visual Analog Scale.

#### 3.6.1 Algorithms and features used

In studies predicting the efficacy of acupuncture using ML, some researches employ multiple ML algorithms simultaneously, while others rely on a single algorithm. Overall, the SVM is the most popular ML algorithm, adopted in 11 out of the 17 reviewed articles. Support vector regression (SVR) and support vector classification (SVC), variants of SVM, are included.

Random forests are an ensemble method that improves prediction accuracy by averaging multiple decision trees. They are used in four articles, while decision trees are featured in one article. Three articles apply logistic regression to evaluate model efficacy. K-nearest neighbors (KNN) are applied in two articles. Four articles utilized deep learning techniques, specifically convolutional neural networks (CNNs), which are suited for analyzing complex imaging data like Magnetic Resonance Imaging (MRI) due to their ability to automatically extract graded features.

Several other methods, e.g., least absolute shrinkage and selection operator (LASSO), similarity-based learning framework, YOLOX, TOOD, XGBoost, ExtraTrees, linear regression, polynomial regression, ridge regression and AdaBoost, are each used only once across the articles. This distribution of ML algorithms employed in acupuncture efficacy prediction studies is summarized in Supplementary Figure 3.

SVM predominates due to suitability for small-sample, high-dimensional medical research. Method diversity reflects the exploratory nature of this emerging field.

Regarding the features analyzed by ML algorithms, MRI is the most commonly utilized sample feature, appearing in seven articles. Questionnaires and scales are the next most frequently used, with six articles incorporating them. Digital Subtraction Angiography (DSA) and electromyography (EMG) utilized in two articles, while Electroencephalography (EEG), Computed Tomography (CT), and Laser Speckle Contrast Imaging (LASCI) are each utilized in one article, as shown in Supplementary Figure 4.

When examining label types used to evaluate acupuncture treatment efficacy, responder classification was the most common, appearing in 11 studies to determine whether patients benefited from treatment. Questionnaire-based scores, e.g., Visual Analog Scale (VAS), the Roland-Morris Disability Questionnaire (RMDQ), and the modified Barthel Index (MBI), were employed in eight studies. Prognostic outcomes were used in 1 study.

#### 3.6.2 Support vector machine (SVM)

SVMs, including SVC and SVR, are widely used in acupuncture efficacy prediction studies. SVM work by identifying the hyperplane that most effectively divides objects into two classes (18). The hyperplane with the maximum margin is selected by SVM (19), and the objects that lie on this margin are called support vectors (20).

Research articles applied SVM in different contexts, and Table 1 listed some studies using SVM. For example, one study employed SVM to predict the efficacy of acupuncture treatment for functional dyspepsia (21). The analyzed features of this study were functional Magnetic Resonance Imaging (fMRI) images, and the outcomes were labeled as effective and ineffective samples based on post-treatment Symptom Index of Dyspepsia scores.

In research involving migraine patients, patients’ pre-treatment gray matter volume was obtained by fMRI (22). The gray matter volume was used as a feature. During the treatment period, the number of migraine days was recorded both before and after treatment. A reduction of 50 percent or more was considered an effective response. SVMs were employed to establish a predictive model for the effectiveness of acupuncture treatment for migraines.

A separate study investigating acupuncture for major depression applied SVM, logistic regression, and random forest (23). EEG features were used to construct the efficacy prediction model. Another analysis focused on predicting acupuncture treatment outcomes in depression using baseline clinical variables, comprising body mass index and anxiety score in self-rating depression scale (24). The performance of SVM, XGBoost, logistic regression, random forest and KNN in predicting acupuncture efficacy were compared.

SVM-based models have also been used to analyze resting-state fMRI data to predict acupuncture efficacy after ischemic stroke. ML also helped identifying the feature brain regions of interest (25). Another study on predicting acupuncture treatment outcomes for upper limb dysfunction after ischemic stroke employed ML models, including SVM (26). DSA diagnostic reports were collected from the stroke patients.

With regard to SVR, one study attempted to predict the efficacy of acupuncture treatment for primary dysmenorrhea (27). Resting-state functional connectivity (rsFC) from fMRI was utilized to establish features for predictive analysis. Another SVR-based article aimed to predict the efficacy and differences between true and sham acupuncture treatments in chronic low back pain using rsFC as features (28). Treatment effectiveness is analyzed for labels, expecting to conduct research using pre-treatment rsFC data.

A combined approach was employed in a migraine without aura study, where SVC was first used to perform a classification analysis on the z-transformed amplitude of low-frequency fluctuation maps (29). SVR then predicted treatment efficacy using significant features identified by the classification step. Similarly, both SVC and SVR were applied to develop the prediction model of acupuncture treatment efficacy in 2 dyspepsia studies (30, 31). SVC was used for response classification, while SVR forecasted symptom improvement.

#### 3.6.3 Deep learning approaches

In studies predicting the efficacy of acupuncture using deep learning, CNNs are prevalent, appearing in as many as four papers. One such paper utilized a multi-view CNN to predict the efficacy of acupuncture treatment for peripheral facial paralysis, comparing its performance with a single-view CNN (32). Two prediction models were constructed, and surface EMG was used as the analytical feature. The features extracted from surface EMG after 4 weeks, along with the Horsfall-Barratt scale, were used as labels. Subsequently, the effectiveness of single-view and multi-view CNNs in predicting acupuncture efficacy was evaluated.

Another study on peripheral facial paralysis explored the mechanisms and efficacy of acupuncture treatment (33). Laser Speckle Contrast Analysis (LASCA) was employed to detect facial microcirculation blood flow for feature extraction, establishing a feature database. CNNs were used for image analysis, and blood microcirculation before and after treatment was analyzed.

In an ischemic stroke study, three deep learning models, including Faster R-CNN, YOLOX and TOOD, were applied for the detection of abnormalities in cerebral vessels happening in patients with upper limb motor complications (34). The detection results were integrated into the treatment efficacy prediction model.

Lastly, a paper utilized deep learning methods to analyze the efficacy of acupuncture treatment for knee osteoarthritis (35). The experiment included a warm needle and moxibustion group and a regular acupuncture group. CT images were used as features before and after treatment, and learning-based analysis was employed to evaluate the efficacy of acupuncture for knee osteoarthritis.

#### 3.6.4 Other machine learning methods

Apart from SVM and deep learning, other ML algorithms have been applied in acupuncture research.

A neck pain research utilized similarity-based learning framework for predicting the efficacy of acupuncture treatment (36). In the study, the Overall Patient-Reported Outcome questionnaire was combined with patient analysis to serve as features for the samples.

Preliminary findings from electroacupuncture treatment studies have also been explored. The LASSO algorithm was employed, referencing two studies on electroacupuncture treatment for chronic low back pain (37). The first study’s data were used to build the model, which was then tested using the second study’s data, verifying the efficacy of electroacupuncture treatment.

### 3.7 Reported outcomes and conclusions on machine learning in acupuncture efficacy prediction

#### 3.7.1 Reported outcomes

In this section, we discuss the effectiveness of ML in predicting acupuncture treatment outcomes. A summary of representative studies is provided below.

A study on chronic low back pain found that pre-treatment rsFC could explain 34% of the variance in pain reduction for real acupuncture and 29% for sham acupuncture (28). In another related study, researchers used data from an initial trial to train a LASSO regression model, which was then applied to clinical data from a second study (37). The model successfully predicted 40% of the variance in Roland Morris disability questionnaire scores.

In studies on migraine without aura, one using SVM models based on MRI features reported an accuracy of 83% to predict headache intensity and frequency of attacks (22), while another study found that baseline activity in the occipital cortex predicted treatment outcomes, with an *R*^2^ of 0.38 for VAS scores and an *R*^2^ of 0.28 for the number of migraine days (29). These results support the use of neuroimaging-based SVM models in treatment prediction.

In the context of neck pain, treatment efficacy was evaluated with a dataset of 794 records, of which 559 were classified as effective (a decline of five points in Northwick Park neck pain questionnaire) and 235 as ineffective (36). The best effectiveness rate exceeded 70%, demonstrating the capability of the applied framework. For primary dysmenorrhea, some pain-related baseline functional connectivity (FC) patterns were shown to predict the VAS change scores and VAS change rate after treatment (27). This study validated the role of SVR in efficacy prediction for the condition.

In ischemic stroke research, an SVM model using resting-state fMRI data predicted motor recovery outcomes with a balanced accuracy of 75.51% and identified eight main brain regions of interest (25). A study on ischemic stroke patients used DSA features and demographics to predict Fugl-Meyer assessment of the upper extremity-based treatment response, achieving 93.6% accuracy with a random forest model (34). Similarly, a model trained on DSA data achieved 88.1% accuracy in predicting MBI outcomes, the highest among three endpoints (26).

For peripheral facial paralysis (32), researchers used surface EMG as features. The results showed that multi-view neural networks performed better than single-view models in facial recognition, with multi-view convolutional networks reaching an accuracy of 88.2% in Horsfall-Barratt scale outcome prediction. Another study utilized CNNs to analyze facial microcirculation during acupuncture and found it could effectively predict treatment outcomes (33).

Several studies on functional dyspepsia reported good predictive performance using SVM. One used rs-fMRI-based functional brain network features, achieving 76% accuracy in identifying responders and *R*^2^ of 0.24 in predicting symptoms relief (21). Another study, using conventional clinical variables as features and treatment response status as the label, achieved approximately 77% classification accuracy and reported *R*^2^ values of 0.446 for the Nepean Dyspepsia Life Quality Index and 0.413 for the Nepean Dyspepsia Symptom Index (31). A more recent study trained models on rs-fMRI and clinical data from 100 patients, reaching 88% accuracy in classifying responders and *R*^2^ of 0.45 in predicting symptoms relief (30).

Regarding acupuncture for depression, a study comparing multiple models (logistic regression, random forest, SVM) with EEG found that nonlinear models performed better, with SVM achieving 84.61% accuracy (23). In another study targeting major depressive disorder using baseline clinical features, XGBoost outperformed other models with 73% accuracy. Both studies used changes in Hamilton depression scale-17 scores to classify treatment responders (24).

Finally, in the field of knee osteoarthritis, one study used deep learning to analyze CT images for efficacy evaluation (35). The significant changes in CT imaging parameters in the test group accompanied by marked improvements in clinical, as well as a higher total effective rate compared to the control group (*p* < 0.05). These suggest that deep learning can effectively assess treatment response using imaging data.

#### 3.7.2 Reported conclusions

As summarized in previous section, ML models have shown potential to predict the efficacy of acupuncture treatments. These preliminary results suggest that those models could be further applied to support clinical decision-making, optimize treatment strategies, and improve resource efficiency.

In studies on low back pain, functional dyspepsia, and depression, ML-supported predictions were found to enhance treatment planning by reducing unnecessary interventions and better allocating resources (21, 23, 24, 28, 30, 31).

Several studies on migraine and ischemic stroke applied ML to enable personalized treatment strategies. Using neuroimaging and clinical data as inputs, these models helped tailor predictions to individual patients and advanced the precision of acupuncture research (22, 26, 34).

The practical use of ML for predicting acupuncture outcomes has also been shown in different clinical conditions. For low back pain and facial paralysis, ML models maintained consistent predictive performance, reinforcing their practical relevance (33, 37).

ML also enhanced treatment precision through the use of neuroimaging and biosignal data. Studies on primary dysmenorrhea, facial paralysis, ischemic stroke, and knee osteoarthritis improved classification of treatment responders by incorporating fMRI, CT scans, and EMG signals as input data (25, 27, 32, 35).

Finally, ML improved the way diagnostic information is analyzed and applied. Research on migraine and neck pain demonstrated that ML can offer new quantitative indicators to evaluate treatment responses and identify patient subgroups (29, 36).

### 3.8 Other applications of machine learning beyond efficacy prediction

Eleven articles explored additional uses of ML on acupuncture research outside efficacy prediction, as summarized in Supplementary Table 1. These studies cover diverse aspects:

- Acupuncture Manipulation Technique Detection: An EEG-based study utilizing functional brain networks reported that the SVM model achieved highest accuracy of 92.14% (38). Another study employing PVDF tactile sensor classified different acupuncture techniques with 88.22% accuracy (39).
- Exploration of Efficacy Factors: One study demonstrated that brain metabolic patterns in the sensorimotor network and default mode network could predict pain relief in patients with primary dysmenorrhea (40). Another using Parkinson’s disease mouse model identified brain regions which correlate with improved motor function (41).
- Acupuncture Prescription Recommendation: A study analyzed clinical records labeled with ICD-10 codes and achieved an accuracy of 79.6% for acupoint recommendation (42).
- Acupoint Sensitization Prediction: A model achieved an average accuracy of 79.2% in prediction (43).
- Acupoint Specificity Identification: One study demonstrated that SVM outperformed the general linear model in differentiating neural responses to stimulation at GB40 and KI3 (44).
- Acupuncture Usage Frequency Prediction: A large-scale analysis of 44,960 patient records used SVM to identify acupuncture patient cohorts, achieving 86.2% accuracy and identifying 101,628 additional patients (45).
- Conduction Effect of Acupoint on Meridians: A study examining propagated sensation along meridians at the Zusanli (ST36) acupoint reported an accuracy of 94.49% in identifying associated factors (46).
- Acupuncture Robotic Point Localization: One study integrated deep learning for acupoint localization, demonstrating high repeatability and adaptability to different skin conditions (47).
- Exploration of Acupuncture Treatment Biomarkers: An ML model used effectively predicted both migraine occurrence and acupuncture treatment outcomes (48).

## 4 Discussion

This scoping review provides an overview of how ML is being integrated into acupuncture research. It summarizes the annual number of publications, the journals in which they appear, and the regions contributing to this growing field. The review also identifies the clinical areas and disease types where ML is applied, as well as the commonly used algorithms, selected features, and their frequency of use.

### 4.1 Trends in machine learning for acupuncture

As the results indicate, the application of ML in acupuncture research was limited before 2019. Overall activity increased notably in the early 2020s. However, this was followed by a significant decline in 2023 and 2024. The decline may reflect a natural plateau after the initial surge, or a shift in focus toward clinical validation with longer study timelines. The low number of three articles for 2024 is likely due to the publication lag. Geographically, most publications originated from mainland China. Contributions from other regions were relatively limited. This geographical concentration was understandable, however, it may have introduced bias, affected the generalizability of findings, and emphasized the need for broader international collaboration.

Several journals published 2 or more articles on this topic, including *Acupuncture Research*, *Frontiers in Neurology*, *Chinese Acupuncture & Moxibustion*, *Chinese Medicine* and *Frontiers in Neuroscience*, indicating sustained academic interest. This pattern suggests that ML-based acupuncture research is gaining recognition across both traditional Chinese medicine and biomedical science communities.

### 4.2 Applications of machine learning in acupuncture

Predicting treatment efficacy is the most extensively studied topic in ML-acupuncture research. The focus on efficacy prediction is driven by several factors: the widespread use of acupuncture, especially for pain management; the variability in patient responses; the reliance on clinical experience and subjective judgment in traditional practice; and the ability of ML to analyze complex data and generate personalized, data-driven predictions. These strengths make ML a valuable approach for enhancing the accuracy and efficiency of acupuncture treatment planning.

Pain relief was the primary focus of studies applying ML to acupuncture. Pain is a subjective experience, but its intensity can be measured with standardized tools like the VAS, allowing for quantifiable data. Changes in pain intensity and frequency can be used as input for training and evaluating ML models. As pain-related disorders are so common in modern society, there is a strong need for reliable methods to predict treatment outcomes. Apart from pain, stroke is evolving as a growing focus for applying ML in acupuncture research these 2 years.

While efficacy prediction is the focus, ML has also been applied to other aspects of acupuncture research, including acupoint selection, technique classification, and the development of acupuncture robotics. These studies are fewer in number but still demonstrate the flexibility of ML in supporting various aspects of acupuncture research.

There are also 8 literature reviews; 5 of them primarily focus on the application of ML or AI in acupuncture research. Further details are provided in Supplementary Table 2. Unlike previous reviews, this study not only examines publication trends and research applications of ML but also offers a deeper analysis of ML methodology. The types of features and labels used in predictive models were discussed, and reported accuracy rates across studies was summarized. In addition, this review incorporates more up-to-date literature to reflect recent advancements in the field.

### 4.3 Machine learning methods for predicting treatment efficacy

In research applying ML methods to predict the effectiveness of acupuncture treatment, models have generally demonstrated favorable predictive performance. Under diagnostic settings, an accuracy or area under receiver operating characteristic curve (AUC) between 0.7 and 0.8 is generally considered acceptable (49). FDA-approved IDx-DR tool is one of the outstanding example, which predicts early diabetic retinopathy from retinal images with 87% sensitivity and 91% specificity (50). For treatment efficacy prediction, a machine learning-based model for statin efficacy achieved accuracy rate of 86.8% (51). This may serve as a benchmark for clinically acceptable predictive performance, which has not yet been reached by most ML models in acupuncture research.

SVM is the most frequently used approach, reported in 11 studies, followed by deep learning method. Compared with complex neural networks, SVM stands out for its ability to work well with small sample sizes, its high interpretability, and its lower tendency to overfit (52). Since recruiting patients for clinical trials can be time-consuming, these advantages make SVM particularly appealing for research in this area. This prevalence emphasizes SVM’s strength in handling high-dimensional biological data and its effectiveness in classification tasks within medical research.

Beyond the choice of algorithm, feature selection and labeling also play a crucial role. Regarding feature selection, imaging data is the most commonly used, followed by data from questionnaires and surveys. However, imaging techniques, especially fMRI, require specialized equipment and can be costly. Therefore, using questionnaires and survey-based features is often a more practical and cost-effective option. The ability of ML in handling diverse data types allows researchers to examine how different features relate to treatment outcomes. Once relevant features are identified, they can provide a foundation for further investigation.

This flexibility suggests that ML could help introduce a wider range of diagnostic indicators in acupuncture research, going beyond traditional methods such as observation, inquiry, and palpation. Research indicates that ML in healthcare can reduce medical costs, support clinical research, and enhance data management efficiency (53). For researchers, it gives more freedom in their studies. For practitioners of TCM, it could offer more tools for tailoring treatments in clinical settings. While SVM, fMRI, and survey-based labels appear frequently, no single method or data type currently dominates. The field remains in an exploratory stage, and many studies apply multiple algorithms in parallel. This ongoing experimentation reflects the evolving nature of the field and the continuing search for effective predictive tools in acupuncture research.

### 4.4 Critical analysis of machine learning integration in acupuncture research

The integration of ML into acupuncture research presents both opportunities and challenges that require careful consideration. This section critically examines the advantages of ML approaches, addresses current limitations, and identifies unmet needs and knowledge gaps in the field.

**Figure 5:**
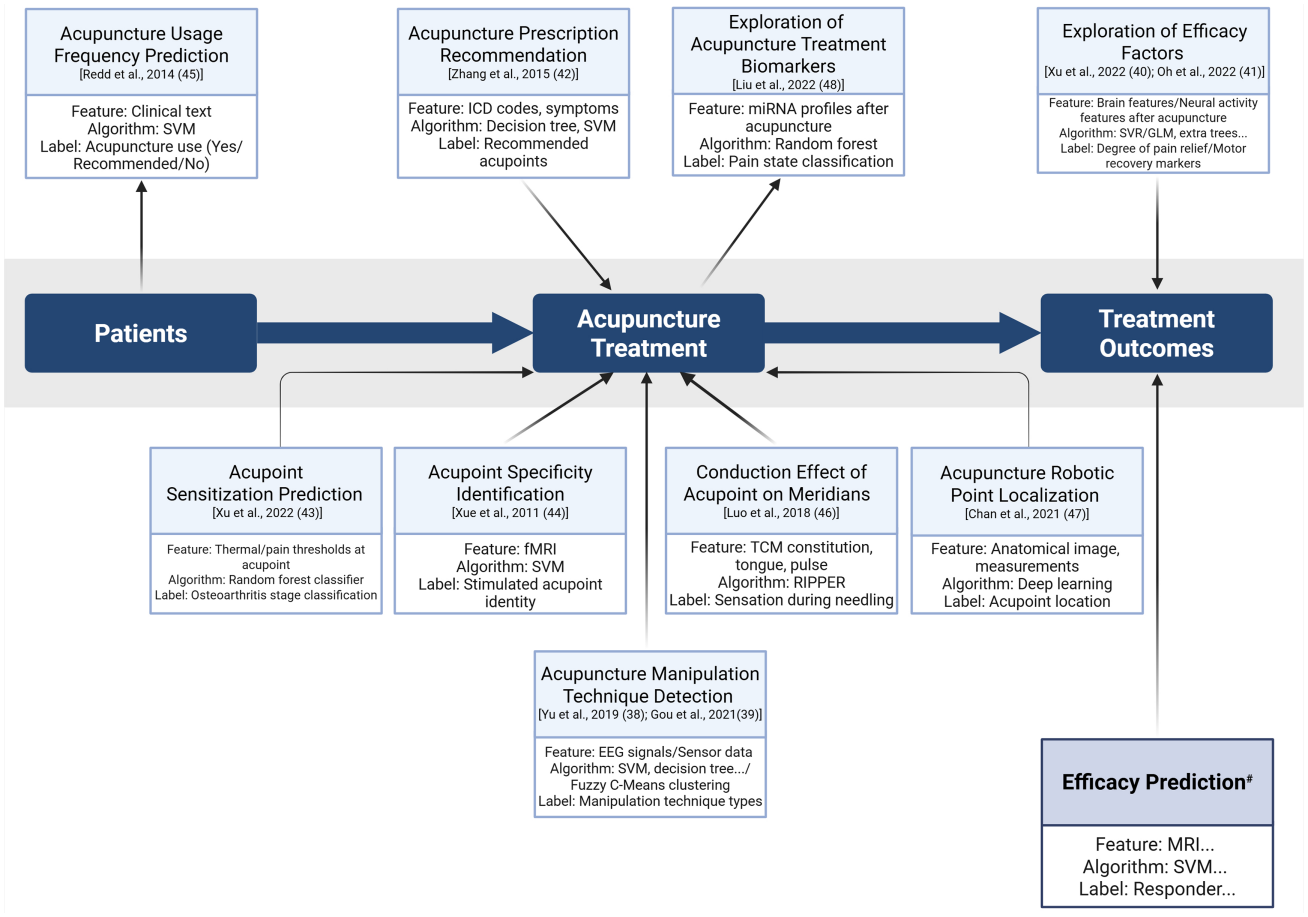
Proposed applications of machine learning in acupuncture clinical settings. The diagram illustrates the integration of machine learning techniques across the clinical workflow, from patient data to acupuncture treatment and subsequent treatment outcomes. The section marked with # is supported by 17 cited references (21–37) (Created in BioRender). EEG, electroencephalography; fMRI, functional magnetic resonance imaging; GLM, generalized linear modeling; ICD, International Classification of Diseases; RIPPER, repeated incremental pruning to produce error reduction; SVM, support vector machine; SVR, support vector regression; TCM, Traditional Chinese Medicine.

The potential applications of machine learning in acupuncture clinical settings are summarized in Figure 5.

#### 4.4.1 Advantages of machine learning-acupuncture integration

ML offers several advantages for acupuncture research. First, ML algorithms can process high-dimensional and multivariate data from neuroimaging to clinical assessments. In our review, 7 of 17 studies on treatment efficacy incorporated fMRI features and demonstrated that brain-based measures can provide objective evaluation of acupuncture related mechanisms.

Second, ML provides personalized prediction of treatment outcomes, addressing the considerable variability in individual responses to acupuncture. Studies on chronic low back pain (28) and migraine (22, 54) showed that baseline neuroimaging patterns could predict 29–40% of treatment response variance. These findings support targeted patient selection.

Third, ML reduces subjective bias in traditional acupuncture practice, which relies heavily on practitioner experience. Classification models in eight studies achieved more than 70% accuracy in predicting treatment responders across multiple conditions, providing objective clinical decision support.

Fourth, ML can identify novel biomarkers and patient response phenotypes. Several studies reported specific brain regions and functional connectivity features associated with treatment success. These results contribute to a more detailed understanding of acupuncture mechanisms.

#### 4.4.2 Limitations and challenges of machine learning-acupuncture integration

Despite these advantages, several limitations restrict current applications of machine learning in acupuncture research. The most significant limitation is the small sample size observed in most studies. Many of the reviewed works included less than 100 participants, which is insufficient for reliable model training and increases the risk of overfitting and poor generalizability.

Across the existing literature, methodological heterogeneity, reflected in differences in study design, sample size, feature selection, and outcome definitions, makes it difficult to draw general conclusions. These issues are compounded by the lack of standardized and openly available datasets, which prevents external validation and slows progress toward reproducible and comparable machine learning models. While some studies report accuracy rates around 70%, it remains unclear whether this level of performance is clinically meaningful without further real-world validation.

Finally, inconsistencies in data types and model evaluation metrics limit the ability to compare findings systematically. Expanding research to include consistent methodologies will be essential to strengthen the evidence base and support clinical translation.

#### 4.4.3 Unmet needs in machine learning-acupuncture integration

Several critical needs must be addressed to advance ML applications in acupuncture research. First of all, the development of standardized datasets with consistent protocols, outcome measures, and data collection procedures is essential for model validation and comparison. Inconsistent data make it difficult to evaluate model performance across studies.

Additionally, multi-center international validation studies are required to assess model generalizability across diverse populations, clinical settings, and variations in acupuncture practice. Single center studies in the current literature limits confidence in the broader applicability.

Lastly, cost effectiveness analyses are necessary to clarify whether machine learning guided treatment selection delivers sufficient value to justify implementation costs, particularly for studies that rely on imaging-based predictors.

#### 4.4.4 Current knowledge gaps

Important knowledge gaps remain in ML research related to acupuncture. The field focuses more on prediction than on mechanistic understanding. The extent to which ML identified biomarkers reflect the therapeutic mechanisms of acupuncture remains unclear.

Also, long term outcome prediction is another unexplored area. Most studies evaluate immediate or short- term treatment effects, and it is not known whether baseline features can predict sustained clinical benefit over longer periods.

The comparative effectiveness of ML guided acupuncture versus traditional experience-based practice has not been evaluated. Evidence showing improved outcomes with ML support will be essential to justify adoption in clinical settings.

These gaps illustrate the early stage of ML research in acupuncture and the substantial work required to realize its full potential in clinical application.

### 4.5 Clinical applications and translation challenges

ML offers strong potential to advance acupuncture practice, but substantial barriers still exist between current research and clinical implementation.

#### 4.5.1 Potential clinical applications

##### 4.5.1.1 Personalized treatment planning

ML can provide direct clinical value by predicting individual treatment response. Studies in our review reported prediction accuracies of 70–88% across conditions such as migraine (22), stroke (25, 34), and functional dyspepsia (21, 30, 31). Baseline fMRI patterns explained 29–40% of outcome variance in chronic low back pain (37), while gray matter volume features predicted migraine treatment responders with 83% accuracy (22). These predictive abilities may enable individualized treatment protocols based on patient characteristics, and more realistic expectation setting through evidence-based outcome forecasts.

##### 4.5.1.2 Optimized acupoint selection

Traditional acupoint selection relies on traditional theory and individual clinical experience, resulting in substantial variability. One study achieved 79.6%accuracy in recommending acupoints based on clinical records (42), suggesting that ML can identify optimal acupoint combinations from large treatment datasets. This capability may help personalize acupoint selection, support less experienced clinicians, and strengthen clinical education by revealing associations between patient presentations and effective acupoint combinations.

##### 4.5.1.3 Disease-specific applications

ML has been applied across several clinical conditions with encouraging results. Models for chronic pain (back pain, neck pain, migraine, dysmenorrhea) may help guide clinical decisions. Stroke rehabilitation models achieved 75–94% accuracy in predicting motor recovery (25, 34), and functional dyspepsia models reached 76–88% accuracy in treatment outcome prediction (21, 30, 31). Depression models reported 73–85% accuracy (23, 24), while facial paralysis studies using EMG achieved 88% accuracy (32). These findings indicate the potential for ML to support decisions regarding patient selection, treatment timing, and treatment intensity.

##### 4.5.1.4 Clinical decision-support systems

The longer-term goal is the development of clinical decision-support systems that integrate multiple functions. These systems may include pre-treatment assessment tools, real-time guidance during treatment, outcome prediction and monitoring, integration with electronic health records, and AI interfaces that explain model reasoning in a way that supports rather than replaces practitioner autonomy.

#### 4.5.2 Translation challenges

Despite promising results, the translation of ML models from research into acupuncture clinical practice faces several challenges. Acupuncture practice often varies between practitioners, in contrast to the standardized protocols used in medical diagnostics. Current ML models often require specific data inputs (neuroimaging, questionnaires) that may not be available in typical acupuncture clinics. The use of electronic health records may offer a more accessible data source to support clinical implementation.

Another major challenge is the personalized nature of TCM. Clinical decision-making in TCM involves pulse diagnosis, tongue examination, and constitutional analysis. These data types have not yet been incorporated into existing ML models. There is a disconnect between what ML models can predict and how practitioners actually make treatment decisions.

Moreover, many practitioners may have limited knowledge toward digital health technologies. They must be able to interpret ML predictions, understand their limitations, and effectively integrate them into practice. Extensive training is needed for clinical adoption.

Last but not least, TCM education emphasizes experiential learning through mentorship and apprenticeship. Successful implementation will require consideration of how ML tools can complement rather than replace traditional diagnostic approaches.

### 4.6 Limitations of this review

This review relied only on PubMed for literature retrieval. PubMed was selected due to its broad coverage of biomedical literature related to the topic. Relevant research published in other databases may have been excluded, for example, computer science conference proceedings. The inclusion of multiple databases, Embase, Scopus, and Web of Science, in future reviews may provide a more comprehensive understanding of the field.

Most of the available studies are published in English, with relatively few in Chinese or other languages, which may introduce a language bias.

### 4.7 Future directions

ML has opened up new pathways for acupuncture research by enabling the analysis of complex, multivariate data. Future research should prioritize the development of standardized datasets, unified outcome measures, and consistent evaluation protocols to address the methodological heterogeneity observed in existing studies. These steps would improve the comparability of findings across studies and support model development.

The methodological heterogeneity and complexity of data suggest that no single ML algorithm is suited for all tasks. The use of hybrid or ensemble frameworks, where various methods are combined, may become an emerging trend. The integration of explainable AI and reinforcement learning may also provide further support. Explainable AI helps make different ML models more interpretable, while reinforcement learning may allow real time and adaptive personalized treatment planning.

Also, prospective and multicenter studies are needed to assess the real-world applicability and clinical utility of ML models, especially those developed to predict treatment efficacy.

Beyond efficacy prediction, ML can support other areas of acupuncture research, including automated acupoint selection and needling technique classification, based on multimodal data (e.g., imaging, biomarkers, and clinical history). These applications can help develop more individualized treatment strategies and better integration of TCM practices with modern evidence-based care.

Finally, expanding research collaborations across geographic regions and disciplinary boundaries will be essential. Engagement between traditional medicine practitioners, biomedical researchers, and computational scientists will lead to more clinically meaningful ML tools that are aligned with real-world healthcare needs.

## 5 Conclusion

ML is currently an emerging field applied across the medical field. Our research indicates that ML applications in acupuncture research have shown encouraging results, with SVM and imaging features commonly used in pain-related studies. Apart from treatment efficacy prediction, ML has been applied to acupuncture technique detection, acupoint prescription recommendation, identification of efficacy-related factors, analysis of acupoint sensitization and specificity, assessment of user behavior, exploration of meridian transmission effects, and the development of acupuncture robotics. The integration of ML into clinical acupuncture practice has potential to enhance the precision, efficiency, and personalization of treatments. However, challenges like small sample sizes, methodological variability, and inconsistent data types limit model generalizability. Future research should focus on larger, multi-center studies with standardized protocols, external validation, and clinical utility assessments to improve performance and support wider adoption in clinical practice.

## Data Availability

The original contributions presented in the study are included in the article/Supplementary material, further inquiries can be directed to the corresponding author.

## Glossary

AI: artificial intelligence
AUC: area under receiver operating characteristic curve
CNN: convolutional neural network
CT: computed tomography
DSA: digital subtraction angiography
EEG: electroencephalography
EMG: electromyography
FC: functional connectivity
fMRI: functional magnetic resonance imaging
KNN: k-nearest neighbors
LASCA: Laser Speckle Contrast Analysis
LASCI: laser speckle contrast imaging
LASSO: least absolute shrinkage and selection operator
ML: machine learning
MRI: magnetic resonance imaging
rsFC: resting-state functional connectivity
SVC: support vector classification
SVM: support vector machines
SVR: support vector regression
TCM: traditional Chinese medicine
VAS: visual analog scale
WHO: World Health Organization
