# Supplementary material for: Integrating machine learning into acupuncture research: a scoping review
Source: Front Neurol. 2026 Jan 14;16:1689061. doi: 10.3389/fneur.2025.1689061 (PMC12848914; doi:10.3389/fneur.2025.1689061)
Supplement: Supplementary file 1 [file Data_Sheet_1.pdf]

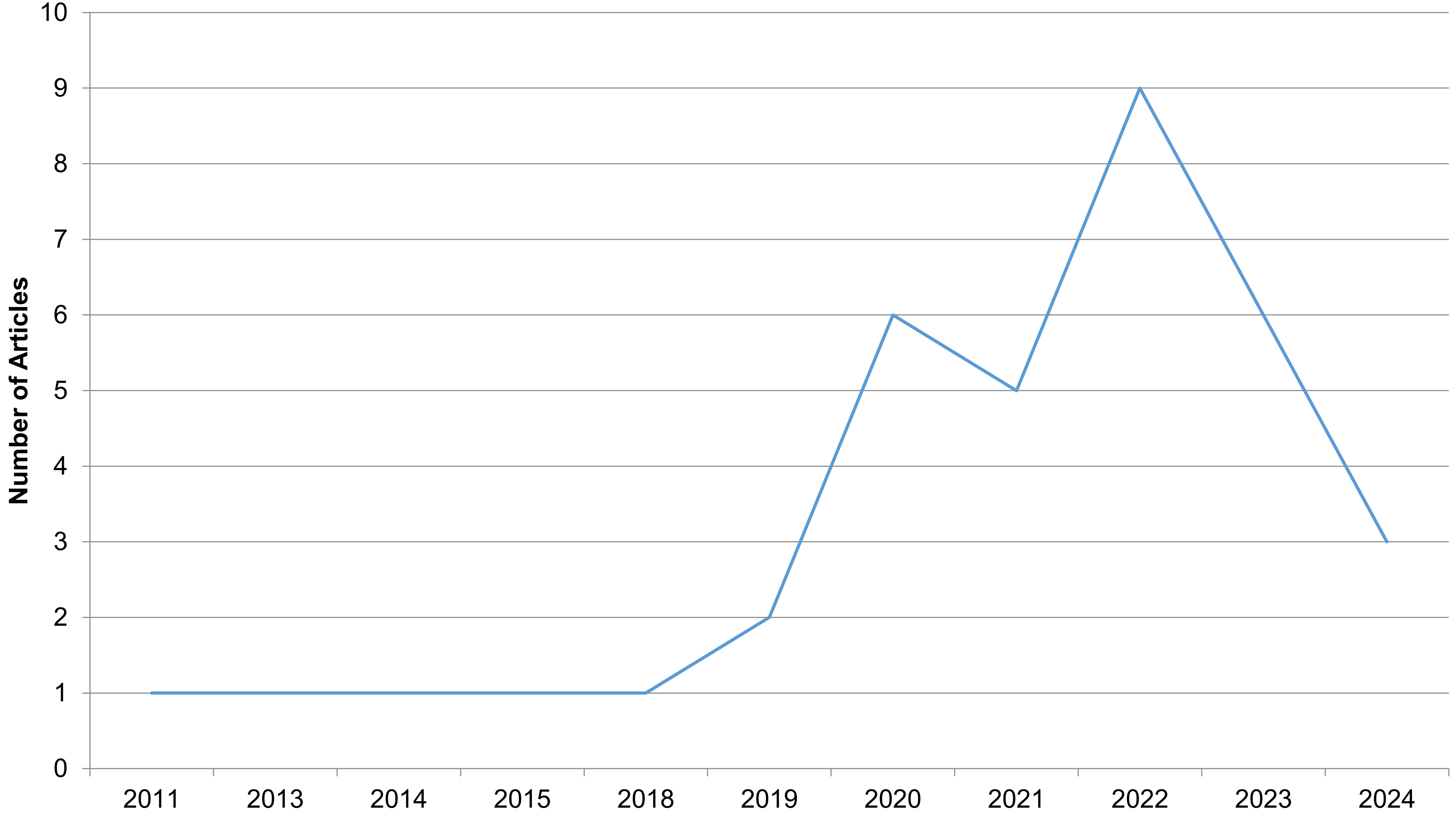

Supplementary Figure 1. Annual number of published articles from 2011 to 2024.

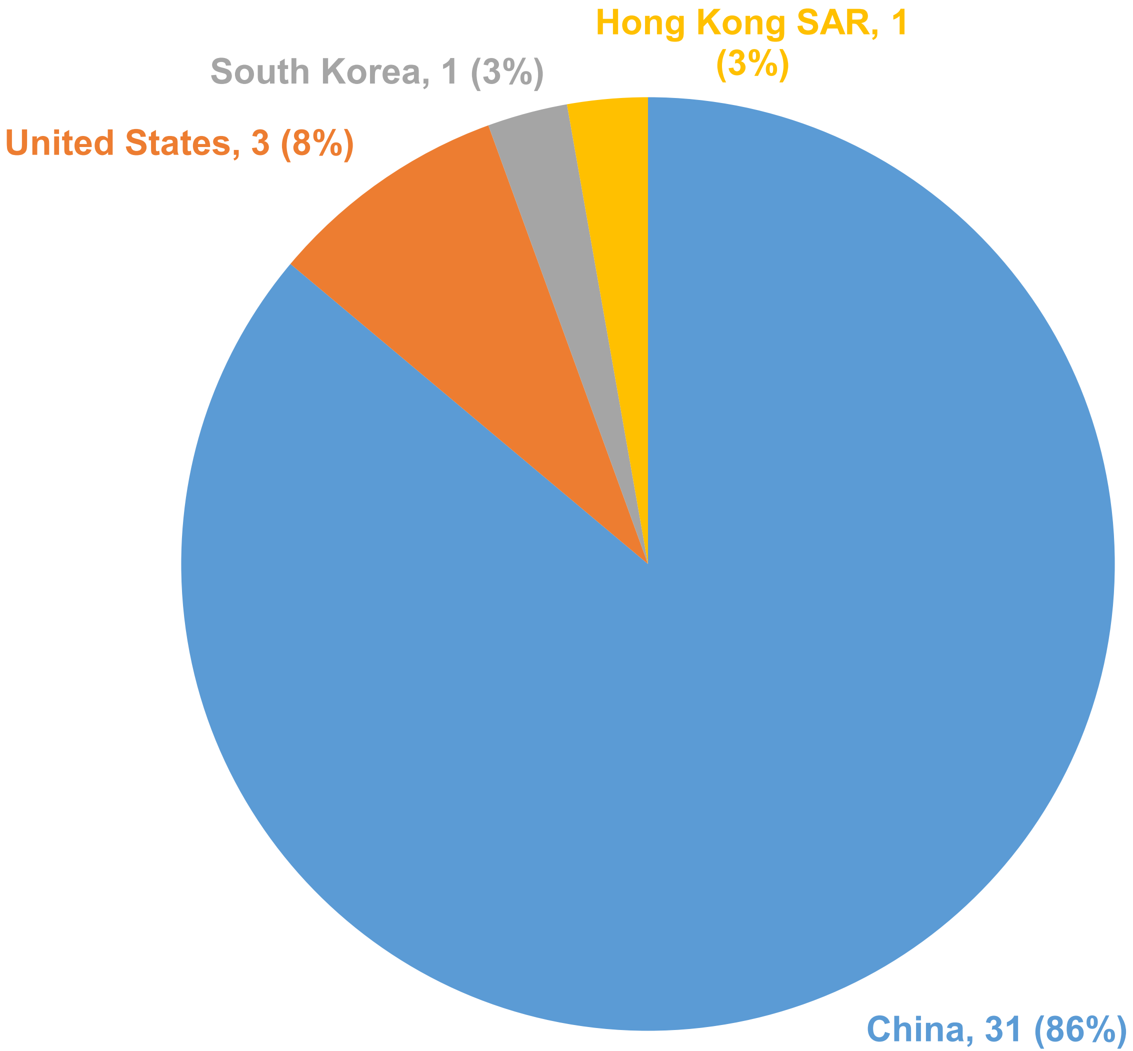

Supplementary Figure 2. Geographic distribution of articles by country or region.

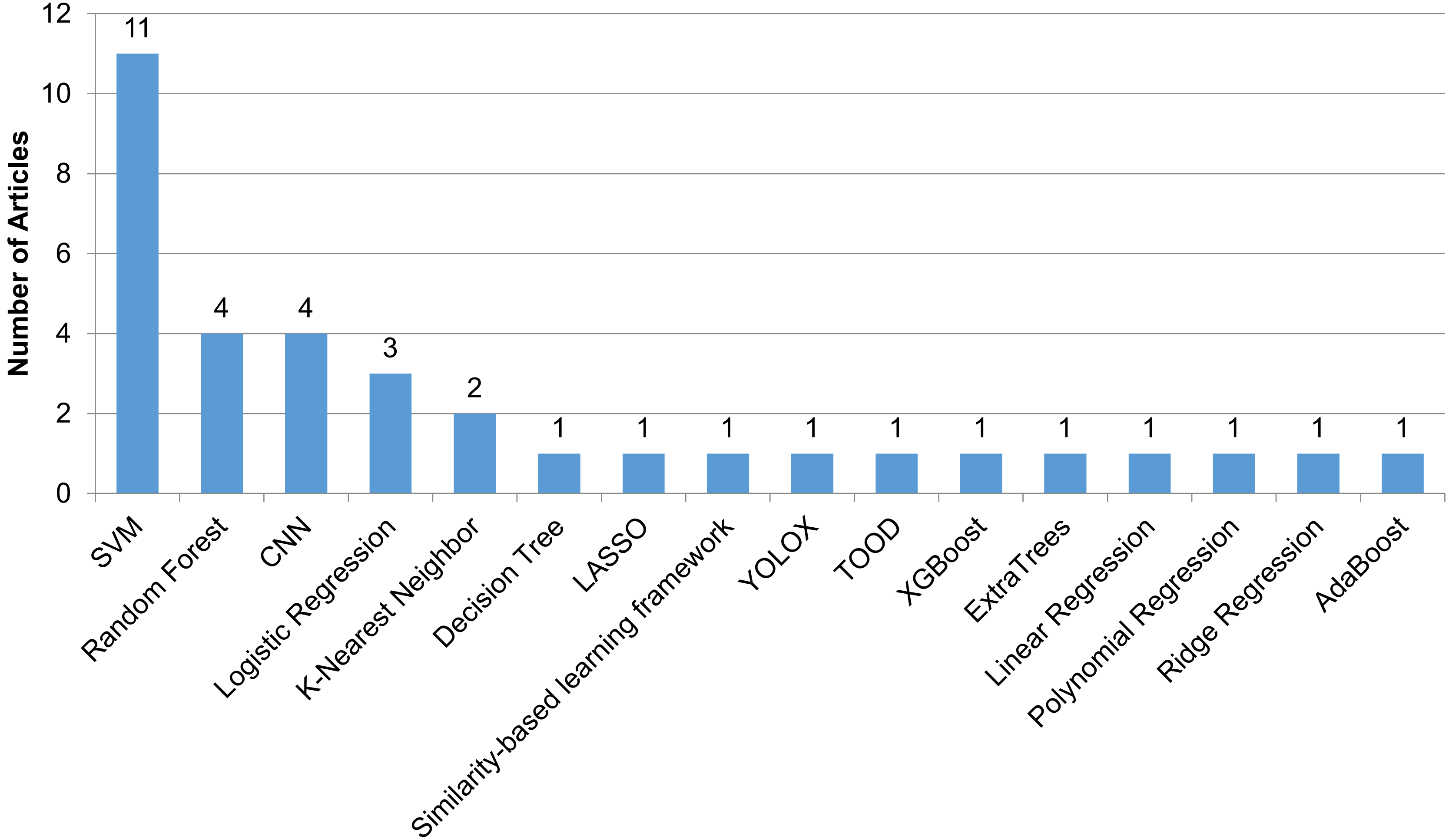

Supplementary Figure 3. Frequency of machine learning algorithms used (some studies used multiple algorithms).

Support Vector Machine (SVM, n=11): Optimal decision boundary classification; Strengths: effective with small samples and high-dimensional data; Limitations: limited interpretability, parameter sensitivity. Random Forest (n=4): Ensemble decision tree method; Strengths: handles non-linearity, resistant to overfitting; Limitations: reduced interpretability. Convolutional Neural Network (CNN, n=4): Deep learning for spatial/imaging data; Strengths: automatic feature extraction, superior imaging performance.; Limitations: requires large datasets, computationally intensive. Logistic Regression (n=3): Binary outcome probability modeling; Strengths: interpretable, efficient; Limitations: assumes linearity, limited with complex patterns. K-Nearest Neighbor (KNN, n=2): Similarity-based classification; Strengths: simple, no training needed; Limitations: computationally intensive, sensitive to scaling. Decision Tree (n=1): Rule-based prediction; Strengths: highly interpretable; Limitations: prone to overfitting, unstable. Other methods (n=1 each): YOLOX/TOOD (object detection), LASSO (feature selection), XGBoost/AdaBoost (ensemble boosting), various regression methods.

SVM predominates due to suitability for small-sample, high-dimensional medical research. Method diversity reflects the exploratory nature of this emerging field.

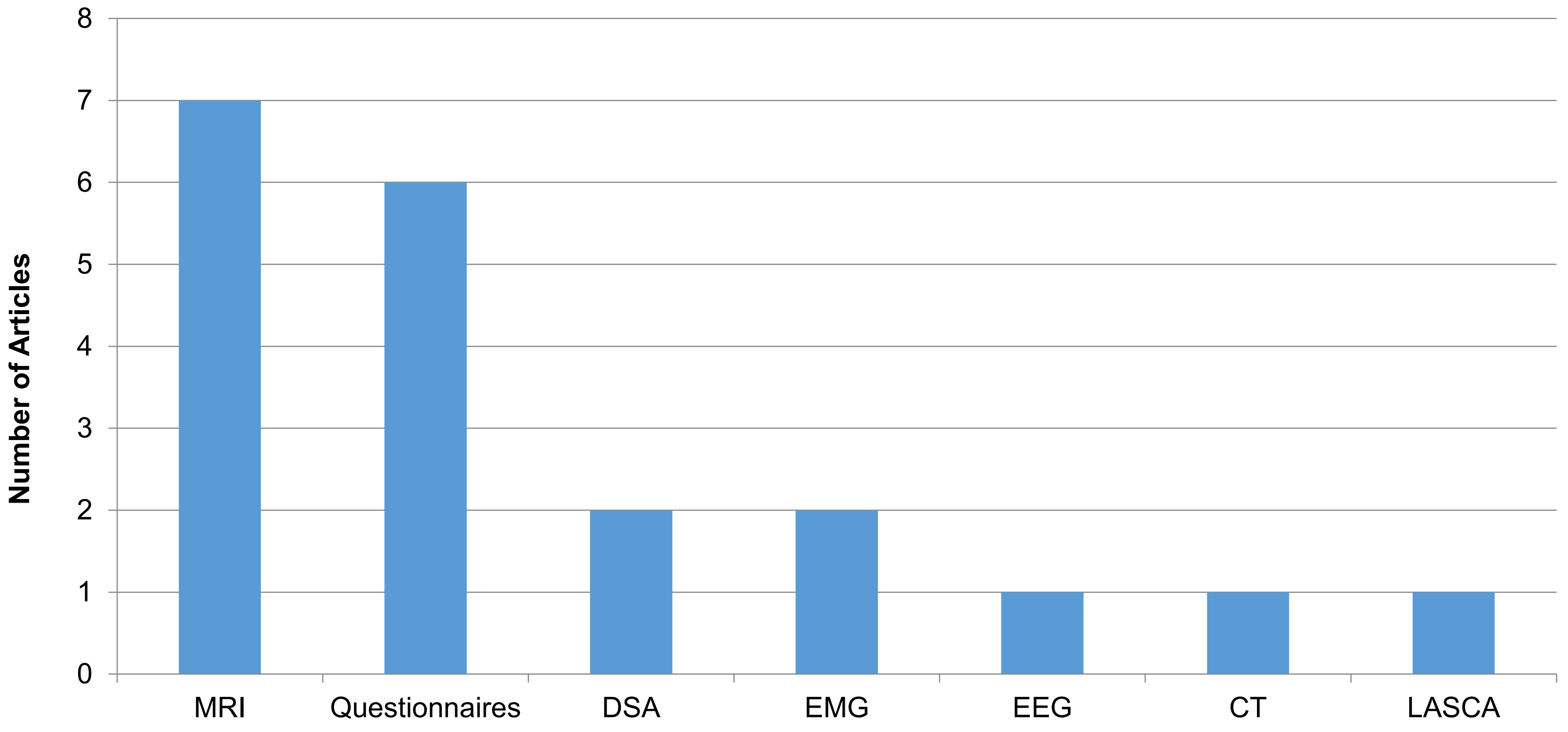

Supplementary Figure 4. Frequency of sample features analyzed by machine learning algorithms (some studies used multiple features).
CT, computed tomography; DSA, digital subtraction angiography; EEG, electroencephalography; EMG, electromyography; LASCA, Laser Speckle Contrast Analysis; MRI, magnetic resonance imaging.
