# Supplementary material for: Integrating machine learning into acupuncture research: a scoping review
Source: Front Neurol. 2026 Jan 14;16:1689061. doi: 10.3389/fneur.2025.1689061 (PMC12848914; doi:10.3389/fneur.2025.1689061)
Supplement: Supplementary file 2 [file Data_Sheet_2.pdf]

Supplementary Table 1. Additional Applications of Research on Machine Learning.

Ref	Research Objectives	Features	Algorithms	Types of ML	Labels	Findings	Applications
(1)[a]	Identify acupoint sensitization in knee osteoarthritis (KOA)	Thermal/pain thresholds at acupoints	Random forest classifier	Supervised learning	KOA stage classification	79.2% average accuracy; 93.3% highest in stage 3 KOA	Acupoint sensitivity: detect changes related to KOA progression
(2)	Distinguish brain responses to different acupoints	Functional MRI activation patterns	Support vector machine (SVM)	Supervised learning	Stimulated acupoint identity	Distinct activation patterns; SVM > General Linear Model	Acupoint specificity: identify distinct neural activation patterns linked to acupoints
(3)	Recommend acupoints using traditional Chinese medicine clinical records	ICD codes, symptoms	Decision tree, support vector machine	Supervised learning	Recommended acupoints	88.2% for ICD, 79.6% for acupoints	Acupuncture prescription recommendation: suggest points from diagnostic records
(4)	Build robotic system for acupoint location	Anatomical images, body measurements	Deep learning	Deep learning	Acupoint location	Accurate and adaptable localization	Acupuncture robots: automate mapping

(5)	Classify acupuncture techniques by electroencephalography (EEG)	EEG signals during manipulation	Support vector machine (SVM), decision tree, naïve Bayes, k-nearest neighbor, logistic regression, neural network, linear discriminant analysis, Takagi-Sugeno-Kang fuzzy system	Supervised Learning/ Unsupervised Learning/ Deep Learning	Technique type (twirling-rotating/ lifting-thrusting)	SVM 92.1% accuracy; twirling-rotating > lifting-thrusting synchronization	Acupuncture technique detection: identify twirling-rotating/ lifting-thrusting from EEG signals
(6)[a]	Detect acupuncture manipulation with sensors	Sensor data (force/time)	Fuzzy C-Means clustering	Unsupervised Learning	Manipulation technique	88.2% overall; >90% for twisting	Acupuncture technique detection: capture mechanical signatures of different manipulations

(7)	Find miRNA biomarkers for migraine acupuncture	miRNA profiles from exosomes	Random forest	Supervised learning	Pain state classification	miR-369-5p, 145-5p, 5010-3p identified	Exploration of biomarkers of acupuncture treatment: identify neuroimaging markers of pain relief
(8)	Predict primary dysmenorrhea relief using PET metabolic data	Brain metabolism features	Support vector regression	Supervised learning	Degree of pain relief	Sensorimotor network and default mode network patterns predicted pain relief	Exploration of efficacy factors: reveal metabolic brain patterns predictive of acupuncture response
(9)	Assess brain effects of acupuncture in Parkinson's disease mice	Neural activity features	Generalized linear modeling, extra trees, random forest, gradient boosting, and XGBoost	Supervised Learning	Motor recovery markers	M1 cortex linked to motor recovery	Exploration of efficacy factors: link brain changes to motor recovery in Parkinson's disease

(10)[a]	Predict meridian sensation in ST36 needling	Traditional Chinese medicine (TCM) constitution, tongue/pulse	Repeated incremental pruning to produce error reduction	Supervised Learning	Sensation during needling	94.5% accuracy; TCM type strongest factor	Exploration of factors affecting meridian conduction effects: predict sensations using TCM features
(11)	Find Veterans Health Administration acupuncture users from records	Mentions of acupuncture in clinical text	Support vector machine	Supervised learning	Acupuncture use: Yes or No	86.2% accuracy; additional 101,628 users identified	Investigation of differences in acupuncture usage: detect users via structured and text records

[a] Articles published in Chinese.

EEG, electroencephalography; ICD, International Classification of Diseases; KOA, knee osteoarthritis; ML, machines learning; MRI, magnetic resonance imaging; PET, positron emission tomography; SVM, support vector machine; TCM, Traditional Chinese Medicine.

Supplementary Table 2. Literature reviews on Machine Learning.

Ref	Research Objectives	Descriptions	Conclusions
(12)[a]	Review machine learning use in acupuncture for prescription, efficacy, and specificity	Summarized machine learning applications including support vector machine, artificial neural network, and decision tree in acupuncture	Machine learning supports standardization and accurate prediction, but faces data limitations
(13)	Review machine learning + neuroimaging to study neuroplasticity from acupuncture	Analyzed machine learning role in decoding neuroimaging changes during acupuncture	Machine learning can reveal complex patterns in brain
(14)[a]	Explore machine learning in acupoint selection, teaching, and efficacy prediction	Reviewed data mining, artificial intelligence teaching tools, and prediction models in acupuncture	Machine learning improves teaching and prediction, but needs better standardization
(15)[a]	Review use of MRI and machine learning for discovering biomarkers of acupuncture analgesia	Summarized neuroimaging (fMRI/sMRI) and machine learning-based studies to identify pain-related brain features	Machine learning + MRI can help identify objective neural markers for acupuncture analgesia, aiding clinical trials
(16)	Review artificial intelligence in acupuncture practice and outcomes	Compared studies on artificial intelligence effectiveness, standardization, and relevance	Artificial intelligence enhances prescription mining and technique quantification
(17)	Analyze artificial intelligence research trends and hotspots in acupuncture	417 papers reviewed for collaborations, keywords, and trends	United States and Harvard lead artificial intelligence-acupuncture research; machine learning is main focus
(18)	Evaluate acupoint choice and outcomes in colorectal cancer postoperative ileus	42 trials analyzed; machine learning model found fundamental acupoints ST36, ST37	Lower He-sea points crucial for gastrointestinal recovery

(19)[a]	Map research trends and challenges in artificial intelligence-acupuncture	511 articles reviewed using CiteSpace and VOSviewer	Interest rising post-2020; limited cross-field collaboration

[a] Articles published in Chinese.

fMRI, functional magnetic resonance imaging

Reference

1. Xu GX, Zhou YM, Sun N, Cui J, Chang XR, Ji LX, et al. [Revealing characteristics and rules of acupoint sensitization phenomena: based on knee osteoarthritis]. *Zhongguo Zhen Jiu*. (2022) 42(1):51-7. doi: 10.13703/j.0255-2930.20210121-0001.
2. Xue T, Bai L, Chen S, Zhong C, Feng Y, Wang H, et al. Neural specificity of acupuncture stimulation from support vector machine classification analysis. *Magn Reson Imaging*. (2011) 29(7):943-50. doi: 10.1016/j.mri.2011.03.003.
3. Zhang G, Huang Y, Zhong L, Ou S, Zhang Y, Li Z. An Ensemble Learning Based Framework for Traditional Chinese Medicine Data Analysis with ICD-10 Labels. *Sci World*. (2015) 2015:507925. doi: 10.1155/2015/507925.
4. Chan TW, Zhang C, Ip WH, Choy AW. A Combined Deep Learning and Anatomical Inch Measurement Approach to Robotic Acupuncture Points Positioning. *Annu Int Conf IEEE Eng Med Biol Soc*. (2021) 2021:2597-600. doi: 10.1109/EMBC46164.2021.9629761.
5. Yu H, Li X, Lei X, Wang J. Modulation Effect of Acupuncture on Functional Brain Networks and Classification of Its Manipulation With EEG Signals. *IEEE Trans Neural Syst Rehabil Eng*. (2019) 27(10):1973-84. doi: 10.1109/TNSRE.2019.2939655.
6. Gou SY, Su C, Wang L, Zhao YN, Chen J. [Recognition system of acupuncture manipulations based on an array PVDF tactile sensor and machine learning]. *Zhen Ci Yan Jiu*. (2021) 46(6):474-9. doi: 10.13702/j.1000-0607.20210155.
7. Liu L, Qi W, Wang Y, Ni X, Gao S, Zhou Z, et al. Circulating exosomal microRNA profiles in migraine patients receiving acupuncture treatment: A placebo-controlled clinical trial. *Front Mol Neurosci*. (2022) 15:1098766. doi: 10.3389/fnmol.2022.1098766.
8. Xu J, Xie H, Liu L, Shen Z, Yang L, Wei W, et al. Brain Mechanism of Acupuncture Treatment of Chronic Pain: An Individual-Level Positron Emission Tomography Study. *Front Neurol*. (2022) 13:884770. doi: 10.3389/fneur.2022.884770.
9. Oh JY, Lee YS, Hwang TY, Cho SJ, Jang JH, Ryu Y, et al. Acupuncture Regulates Symptoms of Parkinson's Disease via Brain Neural Activity and Functional Connectivity in Mice. *Front Aging Neurosci*. (2022) 14:885396. doi: 10.3389/fnagi.2022.885396.
10. Luo X, Li K, Zhang B, Tang C. Influencing factors of the occurrence of propagated sensation effects at Zusanli (ST 36) based on artificial intelligence technology. *Zhongguo Zhen Jiu*. (2018) 38(10):1105-8. doi: 10.13703/j.0255-2930.2018.10.019.
11. Redd D, Kuang J, Zeng-Treitler Q. Differences in nationwide cohorts of acupuncture users identified using structured and free text medical records. AMIA Annu Symp Proc. 2014:1002-9.
12. Yin T, He ZX, Sun RR, Li ZJ, Yu SY, Lan L, et al. [Progress and prospect of machine learning in research of acupuncture and moxibustion]. *Zhongguo Zhen Jiu*. (2020) 40(12):1383-6. doi: 10.13703/j.0255-2930.20191026-0002.
13. Yin T, Ma P, Tian Z, Xie K, He Z, Sun R, et al. Machine Learning in Neuroimaging: A New

Approach to Understand Acupuncture for Neuroplasticity. *Neural Plast*. (2020) 2020:8871712. doi: 10.1155/2020/8871712.
14. Liang J, Han MY, Wang CB, Lu XL, Sun ZR, Yin HN. [Research progress in the integration of machine learning and acupunctology]. *Zhen Ci Yan Jiu*. (2021) 46(6):460-3. doi: 10.13702/j.1000-0607.20210160.
15. Wei XY, Zhang N, Li JL, Shi GX, Wang LQ, Tu JF, et al. [Current studies on biomarkers of acupuncture analgesia using magnetic resonance imaging combining with machine learning]. *Zhen Ci Yan Jiu*. (2021) 46(6):505-9. doi: 10.13702/j.1000-0607.20210161.
16. Wang Y, Shi X, Efferth T, Shang D. Artificial intelligence-directed acupuncture: a review. *Chin Med*. (2022) 17(1):80. doi: 10.1186/s13020-022-00636-1.
17. Zhou Q, Zhao T, Feng K, Gong R, Wang Y, Yang H. Artificial intelligence in acupuncture: A bibliometric study. *Math Biosci Eng*. (2023) 20(6):11367-78. doi: 10.3934/mbe.2023504.
18. Zhang X, Yang W, Shang J, Dan W, Shi L, Tong L, et al. The lower He-sea points playing a significant role in postoperative ileus in colorectal cancer treated with acupuncture: based on machine-learning. *Front Oncol*. (2023) 13:1206196. doi: 10.3389/fonc.2023.1206196.
19. Yu Y, Zhu Z, Li Y, Guo Y, Ren H. [Research status and prospect of the application of artificial intelligence in the acupuncture and moxibustion field based on bibliometric]. *Zhongguo Zhen Jiu*. (2024) 44(8):975-82. doi: 10.13703/j.0255-2930.20231128-k0007.
